# Oral Priming with Replicating Adenovirus Serotype 4 Followed by Subunit H5N1 Vaccine Boost Promotes Antibody Affinity Maturation and Expands H5N1 Cross-Clade Neutralization

**DOI:** 10.1371/journal.pone.0115476

**Published:** 2015-01-28

**Authors:** Surender Khurana, Elizabeth M. Coyle, Jody Manischewitz, Lisa R. King, Glenn Ishioka, Jeff Alexander, Jon Smith, Marc Gurwith, Hana Golding

**Affiliations:** 1 Division of Viral products, Center for Biologics Evaluation and Research (CBER), Food and Drug Administration (FDA), Silver Spring, Maryland, United States of America, 20903; 2 PaxVax, San Diego, CA, United States of America, 92121; 3 PaxVax, Redwood City, CA, United States of America, 94063; Public Health England, UNITED KINGDOM

## Abstract

A Phase I trial conducted in 2009–2010 demonstrated that oral vaccination with a replication competent Ad4-H5 (A/Vietnam) vector with dosages ranging from 10^7^-10^11^ viral particles was well tolerated. HA-specific T-cell responses were efficiently induced, but very limited hemagglutination-inhibiting (HI) humoral responses were measured. However, a single boost of Ad4-H5-Vtn vaccinated individuals with a unadjuvanted licensed H5N1 (A/Vietnam) subunit vaccine resulted in superior HI titers compared with unprimed subjects. In the current study, the impact of Ad4-H5 priming on the quality of the polyclonal humoral immune response was evaluated using a real-time kinetics assay by surface plasmon resonance (SPR). Total binding of serum polyclonal antibodies from the Ad4-H5-Vtn primed groups against both homologous H5N1-A/Vietnam/1194/2004 (clade 1) and heterologous A/Indonesia-5/2005 (clade 2.1) HA1 head domain was significantly higher compared with sera from individuals that received subunit H5N1 vaccination alone. SPR measurements also demonstrated that the antigen-antibody complex dissociation rates (a surrogate for antibody affinity) of serum antibodies against the HA1 of H5N1-A/Vietnam were significantly higher in the Ad4-H5 primed groups compared with those from the unprimed group. Furthermore, strong correlations were observed between the antibody affinities for HA1 (but not HA2) and the virus neutralization titers against the homologous strain and a panel of heterologous clade 2 H5N1 strains. These findings support the concept of oral prime-boost vaccine approaches against pandemic influenza to elicit long-term memory B cells with high affinity capable of rapid response to variant pandemic viruses likely to emerge and adapt to human transmissions.

## Introduction

Pandemic influenza preparedness is largely dependent on the immune status of the human population. In the case of seasonal influenza strains, pre-existing immunity is an important factor in reducing disease severity in most individuals. In the case of avian influenza (H5N1, H7N9, H9N2), there is little or no pre-existing antibody immunity in human populations, which when combined with the higher virulence of some avian influenza virus (AIV) strains can lead to pandemics with high mortality rates. A vaccination strategy that could elicit long term immunity with a probability of cross protection against emerging strains would have great value and impact on global public health. Rapid response to impending pandemics may require alternative vaccine modalities that do not depend on the derivation of vaccine virus via the traditional reassortment and reverse genetics, which often takes several months to accomplish. Heterologous prime-boost approaches have also been evaluated in which different vaccine modalities are employed for priming and boosting [1, 2].

In the current study, we explored the quality of the polyclonal serum antibodies in individuals who were primed three times with replicating Ad4-H5-Vtn (hemagglutinin from A/Vietnam/1194/2004 strain) via the oral route, and were later (3.5–12 months) brought back for a single dose boost with the unadjuvanted licensed H5N1 (A/Vietnam) subunit vaccine from Sanofi Pasteur (90 μg HA/dose). The unprimed placebo group received a single dose of the unadjuvanted H5N1 subunit vaccine [3]. We applied SPR real-time kinetic assays to quantify total antibody binding and serum antibody affinity against recombinant hemagglutinin HA1 (globular head) and HA2 (stalk) domains derived from the H5N1 vaccine strain (A/Vietnam/1194/2004). Technically, since antibodies are bivalent, the proper term for their binding to multivalent antigens like viruses is avidity, but here we use the term affinity throughout, since we do not describe any monovalent interactions. In our microneutralization assay, the Ad4-H5-Vtn primed individuals generated high post-boost neutralization titers against the homologous H5N1 A/Vietnam/1194/2004 (clade 1) strain with >90% seroconversion and seroprotection rates in the groups that received either low or high dose of Ad4-H5-Vtn during the priming protocol (10^7^ and 10^11^ viral particles [VP], respectively), while the unprimed group had only a 40% seroconversion rate (SCR) similar to observed in previous clinical trials with unadjuvanted H5N1 subunit vaccine. Importantly, the Ad4-H5-Vtn primed groups also demonstrated robust cross-clade neutralization. SPR analyses revealed a strong correlation between antibody binding affinity to properly folded functional HA1 globular head (but not to the HA2 stalk) and both homologous and cross-clade H5N1 neutralization titers.

## Materials and Methods

### H5N1 Prime-Boost Study design

**Fig 1:**
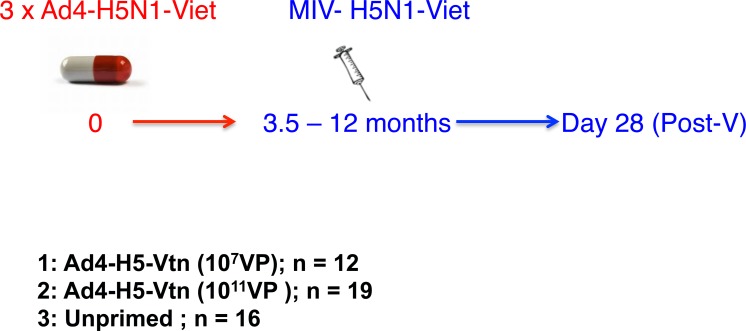
H5N1 prime-boost vaccine trial design. The vaccine study design representing the three groups that were used in the current study and the individual numbers (N) per group is indicated. For more details of the clinical study see Reference 3. Ad4-H5N1-Viet and MIV-H5N1-Viet refer to: Ad4-H5-Vtn is a recombinant, replication competent Ad4, encoding full-length haemagglutinin from influenza A H5N1 virus (A/Vietnam/1194/2004) given orally thrice either as 10^7^ virus particles (VP) or 10^11^ virus particles per dose. MIV-H5N1-Viet is a formalin-inactivated, licensed inactivated H5N1 partially purified subunit vaccine (A/Vietnam, 90 μg HA/dose; Sanofi Pasteur).

The outline of the study was published [3]. In the current study, serum samples from the low dose and high dose Ad4-H5-Vtn primed groups (10^7^ VP and 10^11^ VP, respectively) and unprimed (placebo) group were re-evaluated in the SPR-based assay using HA1 and HA2 proteins (see below). All primed subjects received 3 oral doses of the Ad4-H5-Vtn vaccine 56 days apart followed by a boost with the unadjuvanted licensed inactivated H5N1 subunit vaccine (A/Vietnam, 90 μg HA/dose; detergent treated; Sanofi Pasteur) between 3.5 and 12 month after the last Ad4-H5 prime [3] (Fig. 1). SPR analysis was conducted on pre-vaccination samples (pre-V, Pre-), samples collected 28 days after the third Ad4-H5 prime (Ad4-H5 10^7^ VP, N = 12; Ad4-H5 10^11^ VP, N = 19) (post-P, P-P) and 28 days after the H5N1 subunit vaccine boost (post-V, P-V). None of the subjects in the prime-boost groups seroconverted after the three Ad4-H5-Vtn oral administrations. The placebo group (N = 16) received placebo capsules orally at 56-day intervals followed 3.5–12 months later with a single dose of the H5N1 subunit vaccine. Pre-V and post-V samples were evaluated from the placebo group.

**Ethics Statement:** This trial is registered with ClinicalTrials.gov, number NCT01006798. All samples were de-identified. As described in the publication of the original clinical trial [3], the study was approved by institutional review boards and all subjects signed informed consent forms. The consent form specifically included reference to stored clinical specimens for future research. The study in CBER was conducted with de-identified samples from the original (completed) clinical trial under Research Involving Human Subjects (RIHSC) exemption 03–118B.

### Affinity measurements by Surface Plasmon Resonance (SPR)

Steady-state equilibrium binding of post-H5N1 vaccinated human sera was monitored at 25°C using a ProteOn surface plasmon resonance biosensor (BioRad) [4]. The recombinant HA globular domain (rHA1-His_6_) or HA stalk domain (rHA2-His_6_) for the H5N1-A/Vietnam/1203/2004 influenza virus strain (identical in sequence to the A/Vietnam/1194/2004 vaccine HA) or from A/Indonesia/05/2005 (clade 2.1) was coupled to a GLC sensor chip with amine coupling with 500 resonance units (RU) in the test flow cells. Samples of 60 μL sera at 10-fold & 100-fold dilutions were injected at a flow rate of 30 μL/min (120-sec contact time) for association, and disassociation was performed over a 600 second interval (at a flow rate of 30 μL/min). Responses from the protein surface were corrected for the response from a mock (no coating) surface and for responses from a separate, buffer only injection. Binding kinetics for the human vaccine sera and the data analysis were calculated with BioRad ProteOn manager software (version 2.0.1). Antibody off-rate constants, which describe the fraction of antigen-antibody complexes that decay per second, were determined directly from the serum/plasma sample interaction with rHA1 or rHA2 protein using SPR in the dissociation phase as described before[4] and calculated using the BioRad ProteOn manager software for the heterogeneous sample model. Off-rate constants were determined from two independent SPR runs.

### Neutralization assay

Viral-neutralizing activity was analyzed in a microneutralization (MN) assay in MDCK cells based on the methods of the pandemic influenza reference laboratories of the Centers for Disease Control and Prevention (CDC)[5] with minor modifications provided in updated SOP issued by the CDC. Antibody-neutralization titers by MN were measured against H5N1 vaccine strains of A/Vietnam/1194/2004 (clade 1), A/Indonesia/5/2005 (clade 2.1), A/Turkey/15/2006 (clade 2.2), A/Egypt/3072/2010 (clade 2.2), and A/Anhui/1/2005 (clade 2.3.4). Sera were tested at an initial dilution of 1:20, and those that were negative (<1:20) were assigned a titer of 10. All sera were tested in triplicate, and the geometric mean value was used for analysis.

### Statistical analyses

Differences between groups (p-values) were examined for statistical significance by the multiple comparison adjustment using Bonferroni method. In addition to p-values, the 95% confidence intervals (CIs) of the differences between study groups are also provided. A *p-value* less than 0.05 was considered to be significant. Spearman correlations are reported for the calculation of correlations between off-rate and MN titers combined across all vaccine groups. All statistical calculations were performed using ANOVA.

## Results

### A single H5N1 subunit vaccine boost results in higher HA1 binding antibodies in individuals previously primed with homologous replicating Ad4-H5-Vtn vaccine compared with unprimed individuals

We previously demonstrated high rates of H5 HA-specific CD4 cellular responses following vaccination with the replicating Ad4-H5-Vtn; but HA-specific antibody responses were low; and the impact of pre-existing immunity to the Ad4 virus was overcome in the cohorts receiving the higher dosages[3]. However, we found high HI and neutralization seroconversion rates against H5N1 (A/Vietnam/1194/2004) (Vtn) after a single boosting vaccination with the licensed H5N1 subunit vaccine (90 μg HA/dose) in individuals who had been primed earlier via the oral route with three doses of replicating Ad4-H5-Vtn ranging between 10^7^ and 10^11^ VP (encapsulated) per dose[3]. HI seroconversion rates (SCR) were 100% in the high-dose Ad4-H5 (10^11^ VP) (95% CI, 82–100) and 62% in the low-dose Ad4-H5 prime (10^7^ VP) (95% CI, 32–86). The unprimed control group had a SCR of 36% (95% CI, 17–59) after a single dose of inactivated H5N1 subunit vaccine, confirming the low immunogenicity of the H5N1 subunit vaccine in naïve populations as previously described [6].

In the current study, we evaluated serum samples from the previous clinical trial focusing on the low-dose and high-dose Ad4-H5-Vtn primed groups as well as the unprimed control group after the H5N1 subunit vaccine boost (Fig. 1) for their ability to neutralize homologous and heterologous H5N1 strains from clades 2.1, 2.2, and 2.3. As can be seen in Table 1, the unprimed placebo group gave only 40% SCR against the homologous strain (A/Vietnam) (GMT 60), and the SCR against the heterologous H5N1 strains were minimal (0–20%) with very low GMT (Table 1, bottom row). The low-dose Ad4-H5-Vtn primed group (10^7^ VP) gave 92% seroconversion against A/Vietnam with GMT of 504. Seroconversion rates against clade 2 strains ranged from 50% (A/Indonesia) to 75% (A/Egypt) with GMT ranging between 216–668 (Table 1, top row). The high-dose Ad4-H5-Vtn primed group (10^11^ VP) showed a 94% SCR against A/Vietnam (GMT 1354), and heterologous SCR between 83–89% against the clade 2 viruses with GMT ranging between 694–1102 (Table 1, middle row). Therefore, it was clear that the Ad4-H5-Vtn priming via the oral route, which by itself did not result in significant seroconversion against the vaccine strain, was a powerful prime for a subsequent boost via the intramuscular route with the H5N1 subunit vaccine. Importantly, the prime-boost approach resulted in expansion of the neutralizing capacity against multiple heterologous (clade 2) viruses, including the H5N1 A/Indonesia strain which historically has been difficult to cross-neutralize.

**Table 1:** Geometric Mean and Standard deviations for the end-point microneutralization titers of three vaccine groups against diverse H5N1 virus strains.

Group Name	A/Vietnam(Clade 1)		A/Indonesia(Clade 2.1)		A/Anhui(Clade 2.3.4)		A/Turkey(Clade 2.2)		A/Egypt(Clade 2.2)	
	SCR ^a^	GMT ^b^	SCR	GMT	SCR	GMT	SCR	GMT	SCR	GMT
Ad4 (10^7^)	91.67%	504.17±749.76	50%	215.83±384.4	58.33%	639.17±1461.39	66.67%	237.50±374.73	75.00%	667.50±1449.26
		(10–2560)		(10–1280)		(10–5120)		(10–1280)		(10–5120)
Ad4 (10^11^)	94.44%	1354.44±1170.74	88.89%	694.44±1170.	88.89%	856.67±1168.09	88.89%	1065.56±1546.7	83.33%	1102.22±1544.01
		(20–5120)		(10–5120)		(10–5120)		(10–5120)		(10–5120)
Unprimed	40.00%	60.00±88.80	0.00%	10.00±0.00	0.00%	10.00±0.00	20.00%	42.67±85.98	13.33%	34±79.53
		(10–2560)		(10–5120)		(10–5120)		(10–5120)		(10–5120)

^a^ Seroconversion rate (SCR): Percentage of responders (fraction of subjects) demonstrating MN titers of ≥ 1:40 are shown.

^b^ MN data, expressed as geometric mean titer (GMT), are shown for post-H5N1 vaccination sera collected on day 28 following single H5N1 booster vaccination from the Ad4-H5-Vtn primed and the unprimed control (placebo) group. The range of the individual MN titers are shown in the parenthesis. Titers <20 were assigned a value of 10.

**Fig 2:**
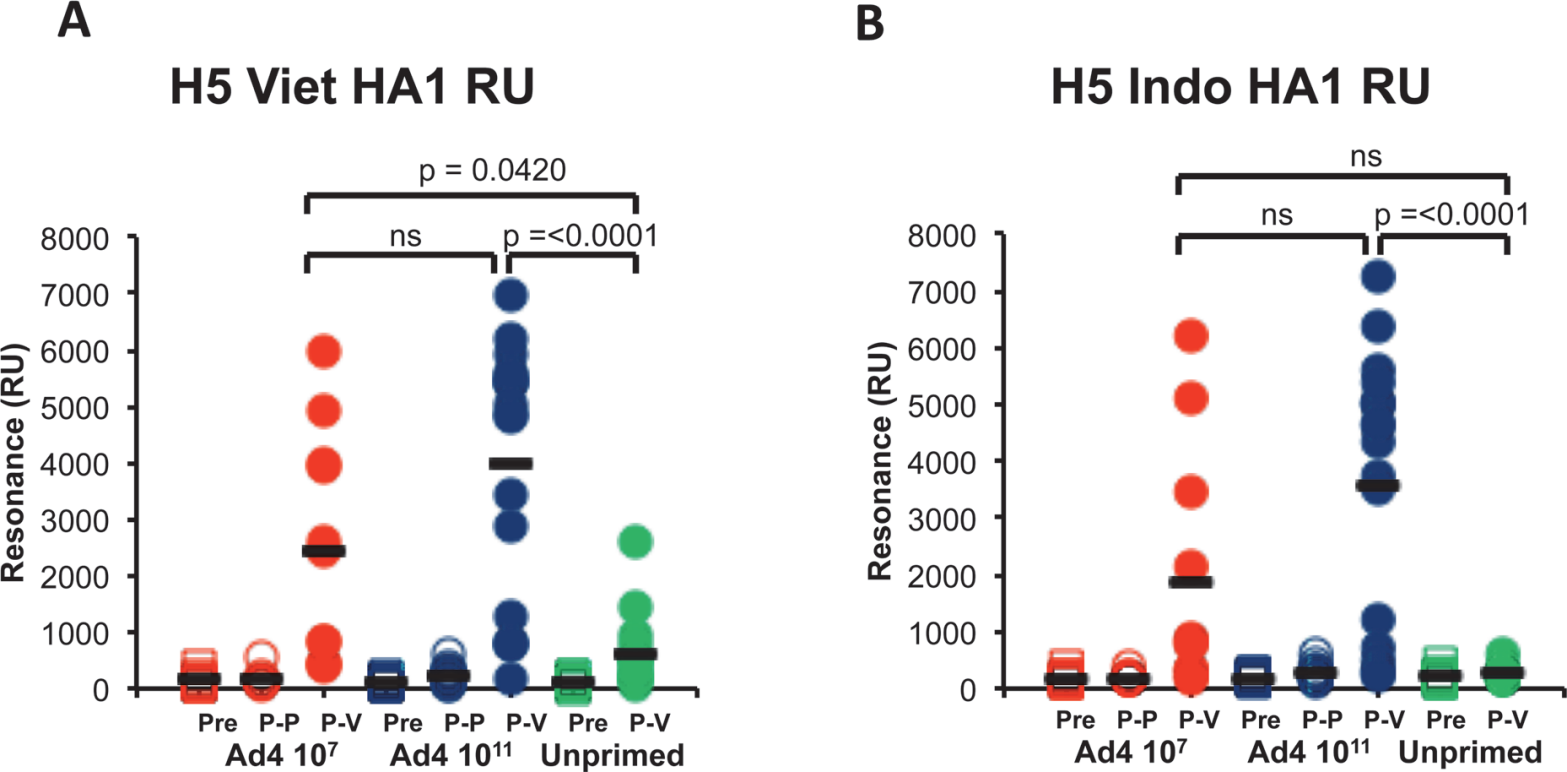
Binding of post-H5N1 vaccination polyclonal human serum to properly folded HA1 proteins from A/Vietnam/1203/2004 and A/Indonesia/05/2005. Steady-state equilibrium analysis of the total binding antibodies in the polyclonal human vaccine sera to properly folded functional H5N1-A/Vietnam/1203/2004 HA1-His_6_ (panel A) or H5N1-A/Indonesia/05/2005-His_6_ (Panel B) was measured by SPR. Each individual post-boost H5N1 serum sample, diluted ten-fold, was injected simultaneously onto HA1 immobilized on a sensor chip through the free amine group and onto a blank flow cell, free of peptide. Maximum resonance unit (Max RU) values for HA1 binding by serum antibodies obtained from multiple individuals from either low dose (10^7^ VP) Ad4-H5-Vtn primed (red circles), high dose Ad4-H5-Vtn (10^11^VP) (blue circle), or unprimed (green circles) on day 0 (Pre), 28 days after the third prime (P-P), and 28 days post boost with 90 μg HA/dose of the Sanofi Pasteur (P-V). Differences between groups (p-values) were examined for statistical significance by the multiple comparison adjustment using Bonferroni method. A *p-value* less than 0.05 was considered to be significant. ‘ns’ represents non-significant (*p = >0.05*).

The goal of the present study was to evaluate the impact of the Ad4-H5 priming on the quality of the antibodies induced against the inactivated H5N1 subunit vaccine boost using a Surface Plasmon Resonance (SPR) based real-time kinetics assay as previously described [4, 7]. For coating of the SPR chips, we captured properly folded recombinant functional HA1 (amino acids 1–320; globular head) and HA2 (amino acids 331–514; stalk) proteins derived from the boosting vaccine strain (H5N1 A/Vietnam/1203/2004) expressed in a bacterial system and purified under controlled refolding conditions [8–10]. Proper folding of the recombinant proteins used in the SPR assay was confirmed by binding to conformation-sensitive, human monoclonal antibodies generated from H5N1-infected survivors as previously described [11]. Total polyclonal antibody binding to rHA1 (max RU) was measured for individual sera from the two prime-boost and unprimed (placebo) vaccine arms. Isotype analysis confirmed that the antibodies that bound the rHA1 and rHA2 proteins were primarily IgG, confirming class switching even in the unprimed placebo group (data not shown). As can be seen in Fig. 2, the post-priming samples (P-P; collected 28 days after the third Ad4-H5-Vtn prime) did not show binding to the rHA1 in SPR. However, serum samples collected 28 days after the subunit vaccine boost (post-V) in the low dose and high dose Ad4-H5-Vtn primed groups gave a broad distribution of binding to H5N1 rHA1 from A/Vietnam/1203/2004 (red and blue circles, respectively). Importantly, the average binding (Max RU) values in these groups were significantly higher than in the unprimed placebo group that only received the subunit vaccine (post-V, green circles) (*p = 0.042* and *p = <0.0001*, with 95% CI of difference 50.74 to 3605 and 1786 to 4916, respectively). Among the two primed groups, the high-dose Ad4-H5-Vtn group had statistically non-significant but higher binding to the A/Vietnam rHA1 protein compared with the low-dose Ad4-H5-Vtn-primed group (Fig. 2A). Importantly, the same binding patterns were observed using heterologous rHA1 protein-coated chips from heterologous A/Indonesia/5/2005 (clade 2.1) strain (Fig. 2B). In that case, no binding to the heterologous rHA1 was measured in the placebo group (Fig. 2B, green circles, post-V). However, serum samples from the two Ad4-H5-Vtn primed groups gave modest to strong binding to rHA1 from A/Indonesia/5/2005 with average max RU values higher in the high-dose prime group (Fig. 2B, blue circles, post-V) compared with the low-dose primed group (Fig. 2B, red circles, post-V) that was statistically not significant. These data also confirm that the rHA1 binding is primarily to conformational epitopes presented on properly folded oligomeric HA1 domains as previously described [11].

### Ad4-H5-Vtn prime—subunit H5N1 boost elicits higher-affinity antibodies against HA1 (but not HA2) compared with antibodies from unprimed individuals

**Fig 3:**
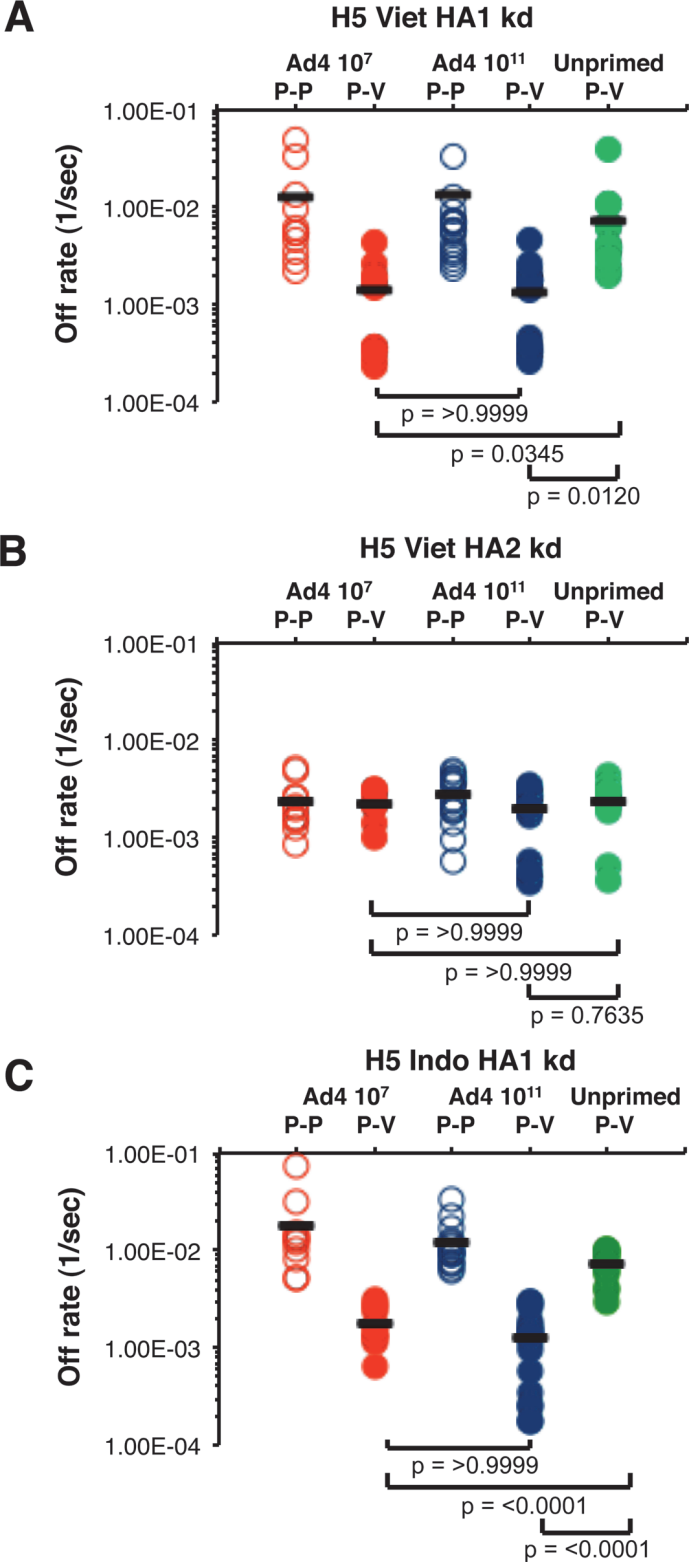
Ad4-H5-Vtn priming enhances antibody affinity (slower off-rates) to H5N1-HA1 (but not HA2) following a single H5N1 subunit vaccine boost. (A-C) SPR analysis of post-H5N1 vaccinated human sera after the H5N1-A/Vietnam/1203/2004 subunit vaccine boost in three vaccine groups was performed with properly folded HA1 (A) or HA2 (B) from H5N1 A/Vietnam/1203/2004 strain, or rHA1 of the heterologous H5N1-A/Indonesia/5/2005 (Clade 2.1) strain (C). Off-rates of polyclonal serum antibodies before (open symbols) or 28 days (filled symbols) after a single H5N1 (A/Vietnam) booster vaccination from low dose Ad4-H5-Vtn primed (red circles), high dose Ad4-H5-Vtn primed (blue circles) or unprimed (green circles) individuals are shown from either 28 days after the third prime (P-P), and 28 days post boost with 90 μg HA/dose of the Sanofi Pasteur vaccine (P-V). Antibody off-rate constants that describe the fraction of antibody-antigen complexes decaying per second were determined directly from the serum sample interaction with rHA1 (1–320) protein or rHA2 (331–480) using SPR in the dissociation phase. Serum antibody off-rate constants were determined as described in Materials and Methods. Differences between groups (*p-values*) were examined for statistical significance by the multiple comparison adjustment using Bonferroni method. *p-values* of less than 0.05 were considered significant.

We have previously demonstrated that it is possible to measure the avidity of antibodies in the polyclonal human sera by measurements of steady-state binding dissociation rates of antigen-antibody complexes using the SPR technology [4, 7, 10, 12, 13]. In the current study, we determined the binding off-rates of serum polyclonal antibodies from all individuals in the three groups, using SPR chips coated with rHA1 or rHA2 from H5N1 A/Vietnam/1203/2004 or A/Indonesia/5/2005 (clade 2.1). As can be seen in Fig. 3A, a significant difference in anti-HA1 polyclonal serum antibody off-rates was found between unprimed and Ad4-H5-Vtn primed individuals (green circles vs. red and blue filled circles) (*p = 0.0345* and *p = 0.012*, with 95% CI of difference -0.01137 to -0.000332 and -0.0108 to -0.001081, respectively). In the Ad4-H5-Vtn primed groups, the antibody binding off-rates to H5N1-rHA1 prior to the subunit H5N1 boost (red and blue open circles) were higher (i.e. lower affinity) than the post boost off-rates by about 10-fold. The post subunit protein vaccination (post-V) antibody off-rates in the unprimed group (Fig. 3A, green circles) were similar to the pre-boost (post-P) polyclonal antibody affinities in the Ad4-H5-Vtn primed groups (Fig. 3A, open red and blue circles). Therefore, these data demonstrate that oral priming with a replicating Ad4-H5-Vtn vector promoted affinity maturation in HA1-specific B cells that could be clearly measured only after the subunit H5N1 vaccine boost. In contrast, when chips were coated with H5N1- rHA2 (Fig. 3B), high-binding affinities were measured for all three groups (dissociation off-rates close to 1 x 10^–3^ per sec) before or after H5N1 subunit vaccination. No statistical differences were found between the anti-HA2 antibody off-rates of primed vs. unprimed vaccinated individual sera. These findings could be explained by the high sequence conservation between the HA2 of H5N1 and seasonal H1N1 strains leading to presence of long term affinity matured memory B cells specific for the H5 HA2 stalk domain in most adults, as described before using whole genome phage display library (GFPDL) [11]. Importantly, the same antibody binding affinity patterns were observed using heterologous rHA1 protein-coated chips from A/Indonesia/5/2005 (clade 2.1) (Fig. 3C). In that case, weak antibody affinity to the heterologous rHA1 was measured in the placebo group (Fig. 3C, green circles, post-V). However, serum samples from the two Ad4-H5-Vtn primed groups generated modest to strong affinity binding antibodies to rHA1 from A/Indonesia/5/2005 with average dissociation rates significantly higher compared with the unprimed group (*p = <0.0001* and *p = <0.0001*, with 95% CI of difference -0.007040 to -0.003952 and -0.007388 to -0.004669, respectively). The HA2 of A/Vietnam/1203/2004 and A/Indonesia/5/2005 are highly conserved and no differences in binding patterns and/or off-rates were found against HA2 derived from A/Indonesia vs. A/Vietnam. In both cases, Ad4Vtn priming did not impact the off rates of serum antibody binding.

These findings suggest that the antibody affinity maturation against different antigenic sites occur independently within the influenza HA and underscores the need to measure antibody affinity separately against each of the antigenic domains within the influenza hemagglutinin, i.e., the HA1 globular domain, which is the most variable among types/subtypes, and the more conserved HA2 stalk domain.

### Higher-affinity anti-HA1 antibodies after Ad4-H5-Vtn prime–subunit H5N1 boost vaccination correlate with broadening of H5N1 cross-clade neutralization

**Fig 4:**
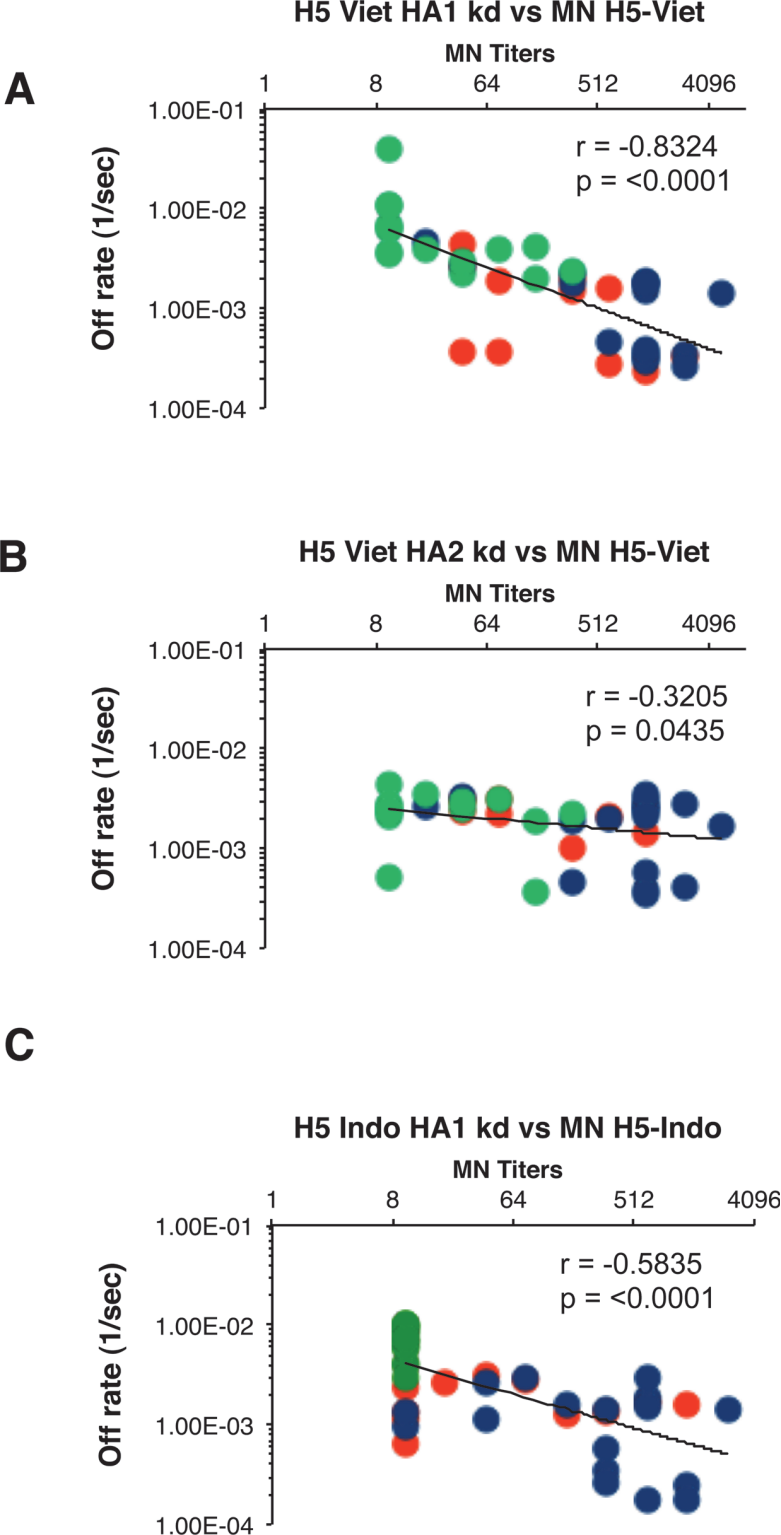
Serum antibody off-rates to H5-Viet-rHA1 (but not rHA2) following heterologous prime-boost strongly correlate with the *in-vitro* neutralizing capacity against the homologous H5 vaccine viruses. Antibody off-rate constants were determined directly from the serum sample interaction with H5N1 rHA1 or rHA2 proteins using SPR in the dissociation phase. SPR analysis of post-boost vaccination human sera from all 3 vaccine groups combined was performed with rHA1 (A) or rHA2 (B) of the H5N1-A/Vietnam/1203/2004 strain, or rHA1 of the heterologous H5N1-A/Indonesia/5/2005 (Clade 2.1) strain (C). Each symbol represents one individual. Serum samples on day 28 following single H5N1 booster vaccination with the subunit H5N1 vaccine (Sanofi Pasteur, 90 μg HA/dose) from the low-dose Ad4-H5-Vtn adjuvanted primed (red circles), high-dose Ad4-H5-Vtn primes (blue circles), or unprimed (green circles) are shown. Antibody affinity of post-H5N1 vaccinated human sera against HA1 (but not HA2) of H5N1-A/Vietnam/1203/2004 correlated with the homologous virus microneutralization titers (MN) against the A/Vietnam/1203/2004 (H5N1). Similarly, polyclonal antibody affinity of post-H5N1 vaccinated human sera against HA1 of the heterologous H5N1-A/Indonesia/5/2005 (Clade 2.1) strain correlated with the microneutralization titers (MN) against the A/Indonesia/5/2005 (H5N1) virus. Spearman correlations are reported for the calculation of correlations between off-rate and MN titers combined across all vaccine groups.

The biggest challenge to pandemic preparedness is the selection of a vaccine modality and composition that is most likely to provide cross-protection against emerging H5N1 (or H7N9) avian influenza strains that may enable human-to-human transmission or arise subsequent to adaptation. Therefore, it was important to determine if the higher affinity of antibodies against H5N1-HA1 (i.e., lower off-rates) found in polyclonal sera from previously Ad4-H5-Vtn primed individuals correlated with the multi-clade H5N1 neutralizing titers (shown in Table 1). The neutralization titers against each H5N1 virus strain were plotted against the antibody off-rates of the individual sera measured against rHA1 or rHA2 derived from H5N1-A/Vietnam/1203/2004 (Fig. 4). A good inverse correlation was found between the rHA1 (A/Vietnam/1203/2004) specific polyclonal sera antibody binding off-rates combined across all groups and the neutralization titers against the H5N1 A/Vietnam/1194/2004 boosting strain (r = -0.8324; *p = <0.0001*) (Fig. 4A). In contrast, the correlation between neutralization titers and rHA2 (A/Vietnam/1203/2004) sera antibody binding off-rates was not obvious with r = -0.3205 (Fig. 4B). Among the vaccine groups, those individuals who received the high dose Ad4-H5-Vtn (10^11^ VP) tend to aggregate in the right-lower part of the correlation curves indicating slowest anti-HA1 antibody off-rates (i.e. highest affinity) and highest neutralization titers (Fig. 4A, blue circles). The serum from individuals receiving no prior priming (green circles) tend to cluster together in the top left part of the curves (low antibody affinity, low neutralization titers). Serum antibodies from individuals in the low dose Ad4-H5-Vtn prime group (10^7^ VP) demonstrated broader distribution of sera antibody off-rates and neutralization titers (red circles). Most importantly, statistically significant inverse correlation was observed between polyclonal serum antibody off-rates against heterologous rHA1 protein-coated chips from A/Indonesia/5/2005 (clade 2.1) and MN titers against the H5N1-Indonesia strain (r = -0.5835; *p = <0.0001*) (Fig. 4C). Thus, priming with Ad5-H5N1 resulted in higher affinity antibodies against both the homologous H5N1-Vietnam/1203/2004 HA1 domain (clade 1.0) as well as heterologous H5N1-A/Indonesia/5/2005 HA1 domain (clade 2.1) that correlated with neutralization titers against the corresponding H5N1 viruses.

**Fig 5:**
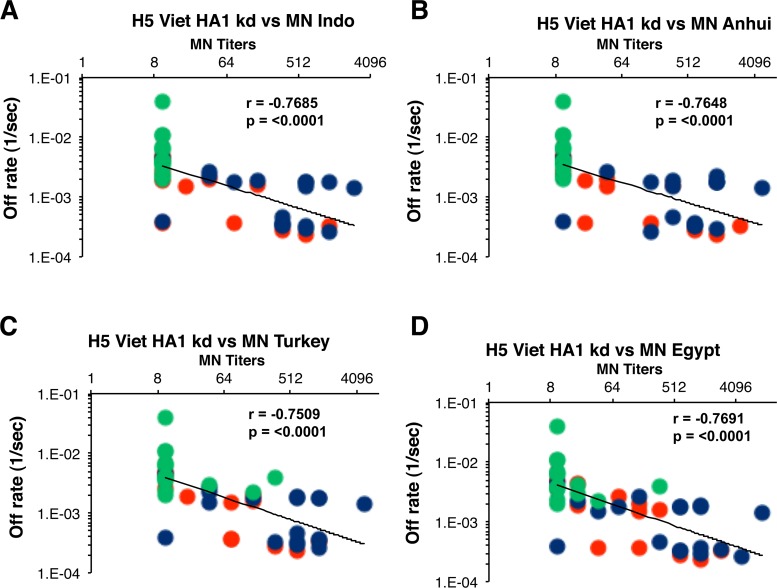
Serum antibody off-rates to H5-Viet-rHA1 following prime-boost strongly correlates with the *in-vitro* cross-neutralizing capacity against diverse H5N1 strains. End-point virus neutralization titers of samples collected after the H5N1 subunit vaccine boost are plotted on the X-axis. Antibody off-rate constants that describe the fraction of antibody-antigen complexes decaying per second were determined directly from the serum sample interaction with rHA1 protein using SPR in the dissociation phase are shown on Y-axis. Serum samples from low-dose Ad4-H5-Vtn primed (red circles), high-dose Ad4-H5-Vtn primed (blue circles) or unprimed (green circles) individuals following H5N1 subunit vaccination are shown. Antibody affinity of post-H5N1 vaccinated human sera against HA1 of H5N1-A/Vietnam/1203/2004 strongly correlated with the *in-vitro* heterologous MN titers against H5N1 clade 2.1- A/Indonesia/5/2005 (A), clade 2.3.4- A/Anhui/1/2005 (B), clade 2.2- A/Turkey/15/2006 (C), and clade 2.2- A/Egypt/NO3072/2010 (D). Spearman correlations are reported for the calculation of correlations between off-rate and MN titers combined across all vaccine groups.

We also correlated the individual binding off-rates against the A/Vietnam/1203/2004 rHA1 and additional cross-clade neutralization titers for all individual samples (Fig. 5). Statistically significant inverse correlations were found between the A/Vietnam/1203/2004 rHA1-specific antibody off-rates and the cross-neutralization titers of H5N1 A/Indonesia (clade 2.1) (panel A, r = -0.7685), H5N1 A/Anhui (clade 2.3.4) (panel B, r = -0.7648), H5N1 A/Turkey (clade 2.2) (panel C, r = -0.7508), and H5N1 A/Egypt (clade 2.2) (panel D, r = -0.7691) with *p = <0.0001*. Interestingly, in both Fig. 4 and Fig. 5, the distribution of serum antibody off-rates and MN titers for individuals in the low-dose and high-dose Ad4-H5-Vtn priming were overlapping, suggesting that even the low-dose priming had a significant impact on the subsequent antibody maturation and virus neutralization (Fig. 4A and Fig. 5, red and blue circles).

Together, our data provide important information on the affinity maturation of the antibodies generated in individuals previously immunized orally with replicating Ad4-H5 vector, then boosted once with a heterologous subunit H5N1 vaccine. The strength and breadth of homologous and heterologous H5N1 virus neutralization correlated with the total binding (Max RU) and affinity of polyclonal sera antibodies binding to the HA1 domain of the boosting H5N1 vaccine strain as determined by SPR.

## Discussion

The current study demonstrated that the affinity of the antibody responses induced against an H5N1 subunit vaccine (A/Vietnam/1194/2004, Sanofi Pasteur, 90 μg HA/dose, administered intramuscularly) in individuals who had previously completed a 3-dose priming regimen with replicating Ad4-H5-Vtn vectors via the oral route was much higher than in unprimed individuals. Importantly, virus neutralization titers strongly correlated with post-boost serum antibody binding to recombinant HA1 from the H5N1 vaccine strain. Furthermore, antibody affinity for rHA1 but not rHA2 (as determined by dissociation rates) strongly correlated with cross-clade H5N1 virus neutralization titers. These data shed light on the advantage of oral priming with replicating viral vectors in generating long-term memory B cells and promoting antibody affinity maturation that may contribute to recognition and neutralization of homologous and heterologous H5N1 virus strains.

The concept of using live replicating adenovirus vectors for oral or intranasal vaccination has been discussed in the past [14, 15]. Moreover, several pre-clinical studies in chimpanzees and non-human primates were conducted with recombinant adenovirus subtype 4, 5, and 7 expressing envelope protein inserts from HIV or hepatitis B virus. In the case of HIV, the intranasal route was found to be more immunogenic than the oral route. Yet in all these studies, a protein boost was required to elicit measurable functional antibodies. Challenge studies suggested either antibody or cell mediated correlates of protection against SHIV challenge [16, 17]. In another study by Lubeck et al., chimpanzees were immunized by sequential oral administrations of Ad7 and Ad4-vectored vaccines containing the hepatitis B surface antigen (HBsAg) followed by HBsAg protein boost. After the booster immunization, anamnestic antibody responses were observed that peaked near 10 mU, a level that in humans is associated with protection from natural infection. After challenge with heterologous HBV, one chimpanzee was protected from acute hepatitis and the other chimpanzee experienced modified HBV-induced disease [18]. These early studies demonstrated the feasibility of using orally (or intranasally) administered recombinant human adenoviruses vectors as a prime for subsequent protein boost that resulted in much stronger antibody responses. However, the impact of the adenovirus-vectored vaccination on the quality of subsequent antibody responses and especially the affinity of the antibodies was not investigated in any of the previous studies.

Maintenance of long-term humoral immunity is provided by a combination of long-lived plasma cells (LLPC) and memory B cells [19–21]. Multiple studies were conducted to better understand the differences between long-lived plasma cells and memory B cells (reviewed by Tarlinton and Good-Jacobson [22]). In a mouse model of West Nile Virus (WNV) infection, it was demonstrated that LLPC generated during the primary response against WNV Domain III of the E glycoprotein were uniformly specific for a dominant neutralizing determinant of the virus, with poor ability to block infection of variant viruses. On the other hand, the memory B cell compartment contained cells with reactivity to viral variants, and after re-stimulation produced some antibodies with higher affinity for the variant virus compared with the inducing viral antigen (*heteroclitic antibodies*). Furthermore, the expanded specificities were associated with germinal center (GC)-derived isotype switched memory B cells [23]. A similar scenario is likely to take place during vaccination or exposure to influenza virus [24]. In the mouse model, it was recently demonstrated that CD4 T-cell help is selective and limiting during the primary B cell response to influenza virus. Preemptive priming of CD4 T-cell help can promote effective and rapid conversion of naïve B cells to mature antibody-secreting cells and improve protection from infection [25, 26]. Therefore, heterologous prime-boost vaccination approaches that prime for HA specific CD4^+^ T cells are likely to drive a strong GC formation leading to maturation of naïve B cells and expansion of early memory B cells with somatic hypermutation (SHM) of the B cell receptors. Strong GC in turn will result in both affinity maturation and repertoire expansion including heterosubtypic specificities as demonstrated in our current and previous studies. Similar to the current study, we have demonstrated a direct correlation between cross-clade neutralization and the development of high affinity antibodies during human clinical trials with adjuvanted vaccines, DNA-prime subunit boost, and LAIV prime subunit boost vaccination strategies [2, 4, 13]. In contrast to the affinity maturation observed in antibodies targeting the HA1 globular head, no significant increase in binding avidity (slower off-rates) was found against the HA2 stalk, and the affinity of HA2-targeting antibodies did not differ between Ad4-H5-Vtn primed and unprimed individuals.

Oil in water adjuvants including MF59 (Novartis Vaccines and Diagnostics) and AS03 (GSK) were shown to significantly improve the humoral immune responses against avian H5N1 influenza and also to induce broad cross-clade neutralizing activities against diverse clades of H5N1 [4, 27–30]. Furthermore, it was demonstrated that an increase in the number of influenza virus specific CD4^+^ T cells after a single dose of MF59-adjuvanted H5N1 vaccine correlated with the rise and long-term maintenance of protective antibody titers against avian H5N1 influenza virus which that reached protective titers only after the second vaccination [31]. More recently, it was demonstrated that T_FH_-like cells with partial phenotype of GC T_FH_ (ICOS^+^, PD1^+^, IL-21^+^) can be found in the blood of humans after vaccination with MF59-adjuvanted influenza vaccine. These cells can exert helper function to influenza virus specific B cells in CD40L- and IL-21- dependent fashion [32]. Their frequencies correlated with the influenza virus specific B cell responses.

In the Ad4-H5-Vtn prime subunit vaccine boost, T-cell responses in terms of IFNγ and IL-2 cytokine production after *in vitro* stimulation of PBMC with rHA or HA overlapping peptide-pools were measured at multiple time points during the priming schedule. They provided strong evidence for dose-dependent increase in the number of positive T-cell cytokine responses but no subset analyses were conducted [3]. It is possible that total PBMC ELISPOT (which includes both CD4 and CD8 cells) may not be optimal for measurement of the most relevant T-cell subset, follicular helper T cells (T_FH_), which are directly involved in interaction with B cells in the germinal centers and induction of somatic hyper mutation/affinity maturation.

In conclusion, the current study provides a better understanding of the increased neutralizing antibody titers and the superior H5N1 heterologous neutralization responses against variant avian H5N1 influenza strains in vaccinated individuals who were previously primed with recombinant Ad4-H5-Vtn orally. It is likely that the orally delivered Ad4 vector expressing the H5-HA leads to the generation of helper T cells in the lamina propria lining the GI tract and possibly early memory B cells that entered the circulation and were available for rapid GC formation after the subunit vaccine boost. Elevated numbers of influenza virus specific T_FH_ cells are also likely to drive the expansion of memory B cells (rather than LLPC) resulting in a more robust long term pool of memory B cells with high affinity BCRs that can recognize variant influenza virus strains not included in the original vaccination [22].

In the case of exposure to novel avian influenza virus strains, a robust recall response of such memory B cells can be the first line of defense against a pandemic influenza threat with diverse H5N1 strains. A further exploration of the impact of oral priming with replicating human adenovirus vectors on memory T_FH_ and B cells is warranted. Such a prime-boost approach may provide a strategy for rapidly inducing immunity in the general population during a pandemic outbreak since the generation of recombinant Ad-HA vectors can be accomplished before the derivation of traditional vaccine strain with high growth/high yield in eggs or mammalian cells. Based on the very strong T-cell responses after priming with the high-dose Ad4-H5-Vtn (>90% response rate[3]), it is expected that priming with the replicating human adenovirus vectored vaccine will be sufficient to elicit the long-term memory B cells after protein boost.
